# Isolation of Specific Genomic Regions and Identification of Their Associated Molecules by Engineered DNA-Binding Molecule-Mediated Chromatin Immunoprecipitation (enChIP) Using the CRISPR System and TAL Proteins

**DOI:** 10.3390/ijms160921802

**Published:** 2015-09-09

**Authors:** Hodaka Fujii, Toshitsugu Fujita

**Affiliations:** Chromatin Biochemistry Research Group, Combined Program on Microbiology and Immunology, Research Institute for Microbial Diseases, Osaka University, Yamadaoka 3-1, Suita City, Osaka 565-0871, Japan; E-Mail: tf1023jp@biken.osaka-u.ac.jp

**Keywords:** ChIP, chromatin immunoprecipitation, enChIP, engineered DNA-binding molecule-mediated ChIP, chromatin, epigenetics, genome function, CRISPR, TAL

## Abstract

Comprehensive understanding of genome functions requires identification of molecules (proteins, RNAs, genomic regions, *etc.*) bound to specific genomic regions of interest *in vivo*. To perform biochemical and molecular biological analysis of specific genomic regions, we developed engineered DNA-binding molecule-mediated chromatin immunoprecipitation (enChIP) to purify genomic regions of interest. In enChIP, specific genomic regions are tagged for biochemical purification using engineered DNA-binding molecules, such as transcription activator-like (TAL) proteins and a catalytically inactive form of the clustered regularly interspaced short palindromic repeats (CRISPR) system. enChIP is a comprehensive approach that emphasizes non-biased search using next-generation sequencing (NGS), microarrays, mass spectrometry (MS), and other methods. Moreover, this approach is not restricted to cultured cell lines and can be easily extended to organisms. In this review, we discuss applications of enChIP to elucidating the molecular mechanisms underlying genome functions.

## 1. Introduction

Genome functions, such as transcription and epigenetic regulation, play essential roles in biological activities. To gain mechanistic insight into the molecular mechanisms underlying specific genome functions, it is necessary to identify molecules associated with the genomic regions of interest. Various experimental approaches have been developed to identify such chromatin-associated molecules. For example, *in vitro* affinity purification of DNA-binding proteins and yeast one-hybrid approaches have been widely used [1]. However, these approaches often neglect the physiological localization of these molecules, leading to detection of interactions that do not occur in the nucleus.

In this regard, for the purpose of biochemical identification of chromatin-associated molecules, it would be straightforward to purify specific genomic regions that retain their molecular interactions. To this end, we developed the insertional chromatin immunoprecipitation (iChIP) technology [2,3,4,5,6,7]. In iChIP, a repeat of the recognition sequence of an exogenous DNA-binding molecule, such as the LexA protein, is inserted into the target genomic region, and a tagged version of the DNA-binding molecule is expressed in cells harboring its recognition sequence. The cells are crosslinked, if necessary, and chromatin is fragmented by sonication or enzymatic digestion. Chromatin complexes containing the tagged exogenous DNA-binding molecule are purified using affinity reagents that recognize the tag. As an alternative approach, we developed “*in vitro* iChIP” [6], in which fragmented chromatin from cells harboring a recognition sequence is incubated with a synthetic or purified form of the cognate DNA-binding molecule, e.g., the recombinant LexA protein, and the target genomic region is then isolated by affinity purification. We hold patents on iChIP (“Method for isolating specific genomic regions”, US patent 8,415,098; Japan patent 5,413,924). After our initial publication of the invention, iChIP has been used by other researchers [8,9,10,11]. Another method to purify specific genomic regions using oligonucleotide probes has been reported [12].

The advent of engineered DNA-binding molecules has changed biological research in a variety of ways. Zinc finger proteins were the prototype engineered DNA-binding molecules [13]. Later, transcription activator-like (TAL) proteins were developed [14], followed recently by the clustered regularly interspaced short palindromic repeats (CRISPR) system [15,16]. These engineered DNA-binding molecules have been used for multiple applications, including genome editing, transcriptional regulation, imaging of genomic loci, genetic screening, and biochemical isolation of specific genomic regions (see review [16]).

In this review, we will discuss applications of engineered DNA-binding molecules to isolation of specific genomic regions for biochemical analysis of genome functions.

## 2. The Principle and Applications of Engineered DNA-Binding Molecule-Mediated Chromatin Immunoprecipitation (enChIP)

### 2.1. Engineered DNA-Binding Molecules

Representative engineered DNA-binding molecules include zinc finger proteins [13], TAL proteins [14], and the CRISPR system [15,16]. Zinc finger proteins were the first molecules to be examined in this regard. TAL proteins were originally discovered as an effector molecule in *Xanthomonas*, a genus of plant pathogenic bacteria. The CRISPR system consists of the Cas9 endonuclease and a guide RNA (gRNA). Introduction of two mutations into wild-type Cas9 abolishes its endonuclease activities but preserves its DNA-binding activity, allowing a catalytically inactive CRISPR system consisting of mutant Cas9 (dCas9) and gRNA to be used as an engineered DNA-binding complex. Zinc finger and TAL proteins recognize their target DNA sequences via protein-DNA interactions, whereas the CRISPR system is targeted via hybridization of gRNA with its target sequence. Because gRNAs targeting different genomic sequences can be generated easily and economically, the CRISPR system is the most flexible and convenient system for creating engineered DNA-binding molecules. However, because the target sites need to contain protospacer adjacent motif (PAM), not all DNA sequences can be targeted by the CRISPR system. Therefore, if there are stringent restrictions on the selection of the target genomic region, other systems (such as TAL proteins) should be used. In this regard, it has been recently reported that the PAM specificities can be altered [17]. Application of this technology to enChIP using CRISPR might allow more flexible selection of the target genomic regions.

### 2.2. Scheme of enChIP

**Figure 1:**
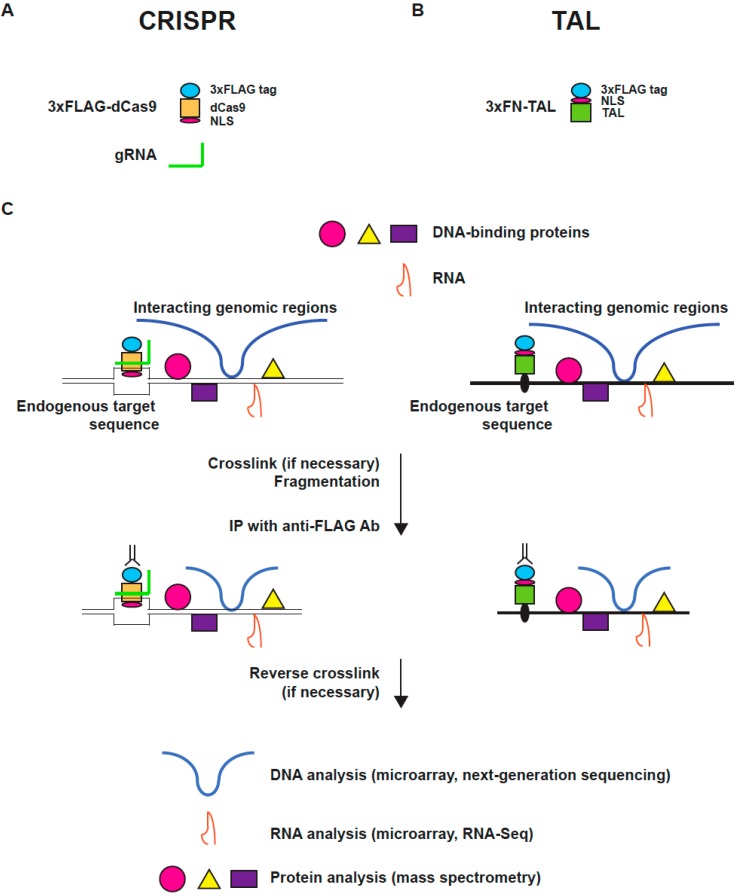
Scheme of enChIP. (**A**) The locus-tagging CRISPR complex consists of tagged dCas9 (in this case, 3xFLAG-dCas9 consisting of the 3xFLAG tag, dCas9, and the nuclear localization signal (NLS) of SV40 T-antigen, as well as gRNA); (**B**) The locus-tagging TAL protein (3xFN-TAL) consisting of the 3xFLAG tag, an NLS, and a TAL protein recognizing the target sequence; (**C**) 3xFLAG-dCas9 and gRNA or 3xFN-TAL targeting the locus of interest are expressed in cells to be analyzed. The cells are crosslinked with formaldehyde or another crosslinker, if necessary, and chromatin is fragmented by sonication or enzymatic digestion. The tagged locus is isolated by affinity purification using anti-FLAG Ab. After reversal of crosslinking (when a crosslinker is used), molecules (proteins, RNAs, or other genomic regions) interacting with the target genomic region are identified by mass spectrometry, NGS, or other methods.

Although iChIP is a powerful approach for the isolation of specific genomic regions, insertion of the recognition sequence of an exogenous DNA-binding molecule into the target genomic region is a time-consuming step that requires significant efforts. The development of engineered DNA-binding molecules led us to consider their use as tags for purification of specific genomic regions, enabling us to dispense with the insertion of recognition sequences of an exogenous DNA-binding molecule, as required in iChIP. Hence, we developed engineered DNA-binding molecule-mediated ChIP (enChIP) using the CRISPR system or TAL proteins. The outline of enChIP is as follows (Figure 1).

(i) Generation of a fusion molecule consisting of an engineered DNA-binding molecule, a tag(s) for affinity purification, and nuclear localization signal (NLS).

(ii) Expression of the fusion molecule into the cell to be analyzed.

(iii) Stimulation of the resultant cells, if necessary.

(iv) Crosslinking with formaldehyde or other crosslinkers, if necessary.

(v) Preparation of chromatin fraction, and fragmentation of chromatin by sonication or enzymatic digestion.

(vi) Affinity purification of the tagged genomic region with antibody (Ab) against the fused tag.

(vii) Reversal of crosslinking, if necessary.

(viii) Identification of proteins associated with the isolated locus using mass spectrometry (enChIP-MS), RNAs using RNA sequencing (enChIP-RNA-Seq), or interacting genomic regions using next-generation sequencing (NGS) (enChIP-Seq).

We have published enChIP using the CRISPR system [18,19] and TAL proteins [6,20,21].

### 2.3. enChIP-MS

enChIP-MS has been used for identification of proteins associated with a given genomic region [18,19,20]. For example, we identified proteins associated with the promoter region of interferon (IFN)-inducible *IFN regulatory factor-1* (*IRF-1*) gene by enChIP using the CRISPR system [18,19]. In that study, we detected chromatin proteins, such as DNA-binding proteins, acetyltransferases, DNA topoisomerases, and histone variants. The fact that we could successfully detect histone deacetylation/corepressor components, whose involvement in IFNγ-induced gene expression has been reported previously [22,23], clearly demonstrated the power of enChIP-MS. In another study, we identified telomere-binding proteins with enChIP using a TAL protein [20], and detected many known telomere-binding proteins. In addition, we identified novel telomere-binding proteins with a variety of localization patterns on telomeres.

### 2.4. enChIP-RT-PCR and enChIP-RNA-Seq

enChIP can be combined with RT-PCR and RNA sequencing (enChIP-RT-PCR and enChIP-RNA-Seq, respectively) for detection of RNAs associated with specific genomic regions. Using a TAL protein recognizing a telomere repeat, we isolated mouse telomeres and detected association of the RNA component of telomerase (*Terc*) by enChIP-RT-PCR [20]. In addition, we used enChIP-RNA-Seq for non-biased identification of RNAs associated with mouse telomeres [21]. We could successfully detect known telomere-associated RNAs, such as *Terc*, *Rmrp*, TERRAs, and scaRNAs. Furthermore, we were able to detect RNAs whose association with telomeres had not been previously reported. To our knowledge, this study was the first report of non-biased identification of RNAs associated with specific genomic regions. enChIP-RNA-Seq will be a useful tool for identification of RNAs associated with a given genomic region.

### 2.5. enChIP-Seq

enChIP can be combined with NGS to perform non-biased identification of genomic regions that interact with a genomic region of interest. In this regard, interactions between genomic regions have been analyzed using fluorescence *in situ* hybridization (FISH) and chromosome conformation capture (3C)-derived methods.

In FISH, distinct genomic regions are labeled with fluorescent nucleotide probes of different colors [24]. Co-localization of these probes suggests interactions of the candidate genomic regions. Drawbacks of this approach include low resolution of the analysis. In addition, non-biased search for interactions between genomic regions using FISH is difficult.

3C was developed in 2002 [25], and several related methods, including 4C (chromosome conformation capture-on-chip), 5C (chromosome conformation capture carbon copy), HiC, ChIP-loop, and ChIA-PET (chromatin interaction analysis with paired-end tags), have been devised (please see details of the methods in [26]). 3C and its derivatives depend on intra-molecular ligation of genomic regions in a chromatin complex. Inclusion of enzymatic reactions such as ligation and digestion with restriction enzymes or other endonucleases can cause detection of signals, which are not physiological interactions but indicate accessibility of the loci.

In this regard, enChIP-Seq enables non-biased identification of interactions between genomic regions. In addition, since enChIP-Seq does not depend on enzymatic reactions, it can be used as a ligation-free alternative to 3C-based methods, especially 4C. On the other hand, as described below, management of off-target binding of engineered DNA-binding molecules would be a potential problem of enChIP-Seq.

## 3. Technical Considerations in Performing enChIP

Detailed protocols for enChIP can be obtained at the authors’ homepage (http://www.biken.osaka-u.ac.jp/lab/microimm/fujii/iChIP_protocols/english.html), bio-protocol publications [27], and our published protocol papers [28,29]. Here, we describe general technical considerations in performing enChIP.

### 3.1. Design of gRNAs and TAL Proteins

enChIP utilizes binding of engineered DNA-binding molecules to the target genomic regions, which may interfere genomic functions [30]. To avoid such potential aberrant effects, we have devised several specific guidelines, as follows:

(1) In the analysis of gene promoter regions, the binding sequences of engineered DNA-binding molecules should be several hundred base pairs upstream (5′) of the transcription start site (TSS). This is because binding of engineered DNA-binding molecules near TSS would directly interfere with the recruitment of transcription factors and RNA polymerases necessary to transcription, and/or disrupt nucleosome positioning around TSS to indirectly inhibit transcription. Because the nature of promoters is different for each gene, it is difficult for us to give precisely how many bases should be separated between the binding sites and TSS. In this regard, increase in the distance between the binding sites of the DNA binder and TSS to decrease in the chance of interference would also decrease in the yields of enChIP. Practically, we would suggest designing binding sites of the DNA binders between −100 and −300 base from TSS and see if the binding might abrogate gene expression. If serious abrogation of gene expression is observed, choose different regions for locus tagging.

(2) By contrast, in the analysis of regulatory regions such as enhancers and silencers with distinct boundaries, engineered DNA-binding molecules can bind to genomic regions directly adjacent to the regulatory regions to be analyzed.

(3) Avoid genomic regions conserved among different species, because those conserved regions often contain functional regulatory sequences such as recognition sites of DNA-binding molecules.

(4) Check the functions of target genomic regions in the presence of engineered DNA-binding molecules. For example, if the target region is a gene promoter region, transcription from the gene should be examined by RT-PCR or other methods.

According to our experience, if these guidelines were observed, it is possible to avoid aberrant effects due to interactions of engineered DNA-binding molecules to the target genomic regions.

Unfortunately, however, it is still possible that the functions of the target genomic regions in the presence of engineered DNA-binding molecules might be abrogated. In this regard, enChIP using CRISPR has an advantage over approaches using TAL or zinc finger proteins, in that plural different gRNAs can be easily and simultaneously tested to select those with minimal aberrant effects.

### 3.2. Transient versus Stable Expression of Engineered DNA-Binding Molecules

Transient as well as stable expression of engineered DNA-binding molecules can be used in enChIP. Transient transfection is convenient when using cells with high transfection efficiency, such as the 293T cell line. On the other hand, stable expression is necessary if the transfection efficiency into the cells to be analyzed is low. Therefore, we have generated a retroviral expression system for enChIP using CRISPR [19]. Plasmids for enChIP using CRISPR can be obtained from Addgene (Table 1). These plasmids include mammalian expression vectors, as well as expression constructs for budding yeast and bacteria.

**Table 1:** Plasmids expressing 3xFLAG-dCas9 available from Addgene.

Plasmid	Species	Selection Marker	Addgene ID #	gRNA Plasmid Addgene ID #	Reference
3xFLAG-dCas9/ pCMV-7.1	Mammalian	-	47948	41824 [31]	[18]
3xFLAG-dCas9/ pMXs-puro	Mammalian	Puromycin resistance gene	51240	retroviral expression vectors of gRNA *	[19]
3xFLAG-dCas9/ pMXs-neo	Mammalian	Neomycin resistance gene	51260	retroviral expression vectors of gRNA *	[19]
3xFLAG-dCas9/ pMXs-IG	Mammalian	GFP	51258	retroviral expression vectors of gRNA *	[19]
3xFLAG-dCas9/ pMXs-I2	Mammalian	hCD2	51259	retroviral expression vectors of gRNA *	[19]
3xFLAG-dCas9/ pTEF1p-CYC1t	Budding yeast	TRP1	62190	43803 [32]	unpublished
3xFLAG-dCas9/ p-bacteria	Bacteria	Chloramphenicol	64325	44251 [30]	unpublished

* Self-inactivating retroviral vectors (pSIR-neo (Addgene #51128), pSIR-GFP (Addgene #51134), pSIR-DsRed-Express2 (Addgene #51135), pSIR-hCD2 (Addgene #51143)) retaining gBlock from gRNA cloning vector (Addgene #41824). See [19] for their construction.

### 3.3. Controls for enChIP Analysis

dCas9 exhibits significant binding to off-target sites [33,34,35,36]. Therefore, it is necessary to perform appropriate controls in enChIP experiments. We suggest the following comparison sets in enChIP analysis to cancel out contamination of off-target sites and other genomic regions.

(1) Compare different conditions for an engineered DNA-binding molecule. This may involve comparisons of the same cell population between different stimulation conditions, e.g., the presence or absence of IFNγ stimulation in our analysis of the *IRF-1* promoter [19], or between different cell types, e.g., T cells and B cells.

(2) Compare several different engineered DNA-binding molecules for each target genomic region. In enChIP using CRISPR, we suggest using several different gRNAs for each target genomic region. Because off-target binding would differ for each gRNA, it is likely that molecules detected commonly by enChIP using different gRNAs represent true positives. To exclude those non-specific molecules associated with dCas9, it would also be prudent to include cells expressing tagged dCas9 but no gRNA.

Needless to say, it is necessary to confirm specific binding of the candidate molecules identified by enChIP using different methods. For example, candidate interacting proteins of a locus of interest can be validated using ChIP analysis.

## 4. Conclusions

enChIP is a powerful and flexible technology for isolation of specific genomic regions for identification of their associated molecules. enChIP can be combined with mass spectrometry, NGS, and other downstream techniques to identify proteins, RNAs, genomic DNAs and other molecules. Use of transgenic animals expressing components of the enChIP system would be an interesting future application of enChIP to developmental studies. enChIP will facilitate understanding of molecular mechanisms of genome functions, including transcription, epigenetic regulation, genomic imprinting, and X chromosome inactivation.
